# Sex Differences in the Primary Prevention of Cardiovascular Diseases in a Dutch Primary Care Setting

**DOI:** 10.5334/gh.1284

**Published:** 2024-01-19

**Authors:** Pauline A. J. Kiss, Alicia Uijl, Estefania Betancur, Annemarijn R. de Boer, Diederick E. Grobbee, Monika Hollander, Charlotte N. Onland-Moret, Miriam C. J. M. Sturkenboom, Sanne A. E. Peters

**Affiliations:** 1 Julius Center for Health Sciences and Primary Care, University Medical Center Utrecht, Utrecht University, The Netherlands; 2 Division of Cardiology, Department of Medicine, Karolinska Institutet, Stockholm, Sweden; 3 Department of Cardiology, Amsterdam University Medical Centers, University of Amsterdam, The Netherlands; 4 The George Institute for Global Health, School of Public Health, Imperial College London, London, United Kingdom; 5 The George Institute for Global Health, University of New South Wales, Sydney, New South Wales, Australia

**Keywords:** sex differences, cardiovascular diseases, primary care, primary prevention

## Abstract

**Background:**

Sex differences in the primary prevention of cardiovascular diseases (CVD) have been shown, but the evidence is mixed and fragmented. In this study, we assessed sex differences in cardiovascular risk factors assessment, risk factor levels, treatment, and meeting of treatment targets, within a Dutch primary care setting.

**Methods:**

Data were obtained from individuals aged 40 to 70 years old, without prior CVD, registered during the entire year in 2018 at one of the 51 general practices participating in the Julius General Practitioner’s Network (JGPN). History of CVD was defined based on the International Classification of Primary Care (ICPC). Linear and Poisson regressions were used to investigate sex differences in risk factor assessment, risk factor levels, treatment, and meeting of treatment targets.

**Results:**

We included 83,903 individuals (50% women). With the exception of glycated hemoglobin (HbA1c), all risk factors for CVD were more often measured in women than in men. Lipid measurements and body mass index values were higher in women, while blood pressure (BP) and HbA1c levels were higher in men, along with estimated glomerular filtration rate (eGFR) levels. Among individuals with elevated BP or cholesterol levels, no sex difference was observed in the prescription of antihypertensive medications (RR 1.00, 95% CI: 0.94–1.06) but women were less likely than men to receive lipid-lowering medications (RR 0.87, 95% CI: 0.79–0.95). Among treated individuals, women were more likely than men to meet adequate levels of blood pressure (RR 1.17, 95% CI: 1.09–1.25) and less likely to meet target levels of cholesterol (RR 0.90, 95% CI: 0.83–0.98).

**Conclusion:**

While women were more likely to have their CVD risk factors measured, they were less likely to be prescribed lipid-lowering medications and to meet target levels. When treated, men were less likely to achieve adequate blood pressure control.

## Introduction

Cardiovascular diseases (CVD) are the leading cause of disease burden in the world [1]. In 2019, an estimated 18.6 million people died from CVD, contributing to 35% of all global deaths in women and 31% in men [2].

Most of the CVD burden can be explained by a set of well-established risk factors [3], including hypertension, dyslipidemia, diabetes, obesity, unhealthy diet, sedentary lifestyle, and smoking [4]. Although these risk factors affect both sexes, sex differences exist in their prevalence [5] and their associations with disease outcomes [4,6,7].

In many countries, general practitioners (GP) are the key healthcare providers to initiate, coordinate, and provide long-term follow-up for CVD prevention [8], serving as the medical gatekeeper for the population at large. Risk factor assessment is essential to detect individuals at high risk for CVD and determine whether they are eligible for interventions. For people without known risk factors, current cardiovascular guidelines recommend assessing the cardiovascular risk every five years in men older than 40 years and women older than 50 years [9]. However, a study from Australia observed that women were less likely than men of similar age to have their CVD risk factors measured [10]. Furthermore, several studies have shown that women may receive a delayed and less intensive treatment compared with men [10,11,12]. Despite growing awareness of their CVD burden, women are still less likely than men to receive cardiovascular therapies and to meet treatment targets as recommended by guidelines, with the biggest shortfalls occurring in younger women [9,13].

Whilst there have been major advances in the implementation of recommended cardiovascular prevention in primary and secondary care [14,15], a full understanding of sex differences across multiple aspects of CVD prevention is lacking in the present literature. Evidence of sex differences per age group exists, but is scarce, though relevant to study, since women are likely to develop CVD later in life, with a first CVD event occurring 5 to 10 years later than men [16]. Increased awareness of sex differences in the prevention of CVD will help to improve CVD preventive strategies, strengthen the implementation of individual risk-based prevention in primary care and promote a better cardiovascular health for everyone.

In this study, we aim to describe primary prevention practices of CVD in a primary care setting in the Netherlands, and assess sex differences in cardiovascular risk factors assessment, risk factor levels, treatment and meeting of treatment targets across different age groups.

## Methods

### Source and study population

This study is a descriptive cross-sectional study using electronic health records (EHR). Data were obtained from the Julius General Practitioners’ Network (JGPN). JGPN is a data source that includes pseudonymised routine healthcare data from a large ongoing dynamic population of individuals registered with the participating general practices from the city of Utrecht and its surrounding area, as detailed elsewhere [17]. Briefly, JGPN was initiated in 1996 in the region Utrecht by the collaboration of six general practices and the University Medical Center (UMC) Utrecht and had the objective to make routine care data accessible for clinically relevant research [17]. All individuals registered with one of the JGPN practices that did not choose to opt-out to data sharing for research, are included in the JGPN source population. The JGPN population is considered representative of the Dutch population with regard to sex and age [17]. In 2018, the network consisted of 82 participating centers, with a total of 292,046 registered individuals.

The study population comprised of 83,903 individuals aged 40–70 years at 01/01/2018, who were registered at one of 51 general practices, and had follow-up data available for the entire year of 2018. We excluded individuals with a CVD event recorded in the EHR before the date of the first measurement for a CVD risk factor recorded in 2018. In absence of measurement in 2018, we excluded individuals with a CVD event recorded before 2018. The definition of history of CVD can be found in Supplementary Table 1.

Sex differences in cardiovascular risk management were assessed on four aspects: risk factor assessment, risk factor level, risk factor treatment among those with elevated levels, and risk factor control among those treated. The descriptive analyses on risk factor assessment and level were conducted in the above-described study population. Risk factor treatment was assessed among individuals with elevated levels; that is, those with a systolic blood pressure (SBP) / diastolic blood pressure (DBP) ≥ 140/90 mmHg or low density lipoprotein cholesterol (LDL-c) ≥ 2.6 mmol/l, recorded in 2018 (n = 8,210 and 10,405, respectively), following Dutch guidelines for cardiovascular risk management [18]. Risk factor control was assessed in individuals with at least one prescription for either blood pressure-lowering medication or lipid-lowering medication in the six months prior to their first measurement of blood pressure or cholesterol in 2018 (n = 6,569 and 3,553, respectively). In doing so, we ensured that the individuals were prescribed medication that could influence the control of the risk factor levels. Graphical depictions of the study design of each analysis can be found in Supplementary Figures 1–3.

### Data extraction

Data on cardiovascular risk factors, history of cardiovascular events and drug prescriptions were extracted from the medical records. The first available measurement in 2018 of the following nine risk factors were analysed in this study: physical examination (SBP, DBP, body mass index (BMI)), lipid measurements (total cholesterol (TC), LDL-c, high-density lipoprotein cholesterol (HDL-c), triglycerides (TG)) and biomarkers (glomerular filtration rate (eGFR), average glucose level (HbA1c)). Data on drug prescriptions for antihypertensive drugs (Antihypertensives, Diuretics, Beta blocking agents, Calcium channel blockers (CCB), Angiotensin-converting enzyme inhibitors (ACE-i), Angiotensin II receptor blockers (ARB)) and lipid-lowering drugs (HMG CoA reductase inhibitors, fibrates, bile acid sequestrants, nicotinic acids and derivatives) were identified using the anatomical therapeutic chemical classification (ATC) system. The definitions and codes for medical history and drug prescriptions can be found in Supplementary Table 1.

### Outcomes

A risk factor was considered to be assessed if a measurement was registered in 2018 in the data source. The risk factor levels were assessed at the first measurement in 2018. Risk factor treatment was assessed in the subcohort by the presence of at least one prescription for blood pressure-lowering medications or lipid-lowering medications in 2018. In the analyses for risk factor control, we assessed the proportion of treated individuals that attained adequate levels of SBP and LDL-c, according to Dutch guidelines [18].

### Statistical analysis

Baseline characteristics were analysed by sex and categorised in three age groups: 40–50 years, 50–60 years, 60–70 years. Poisson regressions with robust standard errors were used to estimate age-adjusted women-to-men risk ratios (RRs) and 95% confidence intervals (CIs) for the associations between sex and cardiovascular risk factor assessment, treatment prescription, and risk factor control. For the assessment of risk factor levels, a linear model was used to obtain age-adjusted women-to-men mean differences (MDs). The normality assumption was checked for each risk factor and log transformations were applied for TG and HbA1c, as they followed non-normal distributions. An interaction term for sex and age (as categorical variable) was added to all analyses to assess whether the effect of sex on the outcome varied with age. Complete cases were used for all analyses. All preprocessing and analyses were conducted with the R software version 4.1.2. Statistical code used in this study is available on request.

## Results

### Study population

We included 83,903 individuals (50.4% women) with an average age of 53 years old (Standard Deviation (SD) = 8.4) (Table 1 and Supplementary Table 2).

**Table 1:** Baseline table of the study population.


n (%)	MISSING, n (%)	OVERALL	WOMEN	MEN

		83 903 (100)	42 262 (50.4)	41 641 (49.6)

**Age (mean (SD))**	0 (0)	52.8 (8.4)	53.0 (8.6)	52.5 (8.3)

Visits (median (IQR))*	58 200 (69.4)	2.0 (1.0–3.0)	2.0 (1.0–3.0)	2.0 (1.0–3.0)

Risk factors (mean (SD))**				

Systolic Blood Pressure (mmHg)	66 642 (79.4)	136.2 (19.0)	134.7 (19.4)	137.9 (18.2)

Diastolic Blood Pressure (mmHg)	66 660 (79.5)	82.9 (11.2)	82.0 (11.0)	83.9 (11.2)

Cholesterol (mmol/L)	68 470 (81.6)	5.14 (1.1)	5.26 (1.1)	5.01 (1.1)

Triglycerides (median (IQR); mmol/L)	68 522 (81.7)	1.40 (1.0–2.0)	1.30 (0.9–1.8)	1.50 (1.1–2.2)

HDL cholesterol (mmol/L)	68 519 (81.7)	1.32 (0.4)	1.44 (0.4)	1.18 (0.3)

LDL cholesterol (mmol/L)	68 986 (82.2)	3.10 (1.0)	3.15 (1.0)	3.03 (1.0)

Body mass index (kg/m^2^)	72 674 (86.6)	29.0 (5.8)	29.4 (6.4)	28.6 (5.1)

HbA1c (median (IQR); mmol/mol)	78 338 (93.4)	50.0 (42.0–58.0)	49.0 (42.0–57.0)	50.0 (42.0–60.0)

eGFR (median (IQR); mL/min/1.73 m^2^)	65 542 (78.1)	83.0 (73.0–95.0)	82.0 (72.0–94.0)	85.0 (75.0–96.0)

**Elevated risk factor levels (%)**				

LDL-c ≥ 2.6 mmol/L	/	12.4	13.5	11.3

SBP/DBP ≥ 140/90 mmHg	/	9.8	9.9	9.7

Medications (%)***				

Lipid-lowering drugs	/	8.0	7.4	8.6

Statins	/	7.9	7.3	8.5

Antihypertensive drugs	/	14.8	15.9	13.7

ACE inhibitors	/	5.8	5.2	6.4

ARB	/	3.3	3.6	3.0

Beta blockers	/	5.6	6.5	4.6

Calcium chain blockers	/	4.3	4.2	4.5

Diuretics	/	5.6	6.3	4.9


* Refers to the number of weekly aggregated risk factor measurements in 2018. ** The first measurement in 2018 was selected per id. *** Refers to the number of individuals with at least one prescription in the year 2018. HDL cholesterol = high-density lipoprotein cholesterol; LDL cholesterol = low-density lipoprotein; HbA1c = glycated hemoglobin; eGFR = estimated glomerular filtration rate; ACE inhibitors = angiotensin converting enzyme inhibitor; ARB = angiotensin receptor blocker; SD = standard deviation; IQR = interquartile range.

### Risk factor assessment

**Figure 1:**
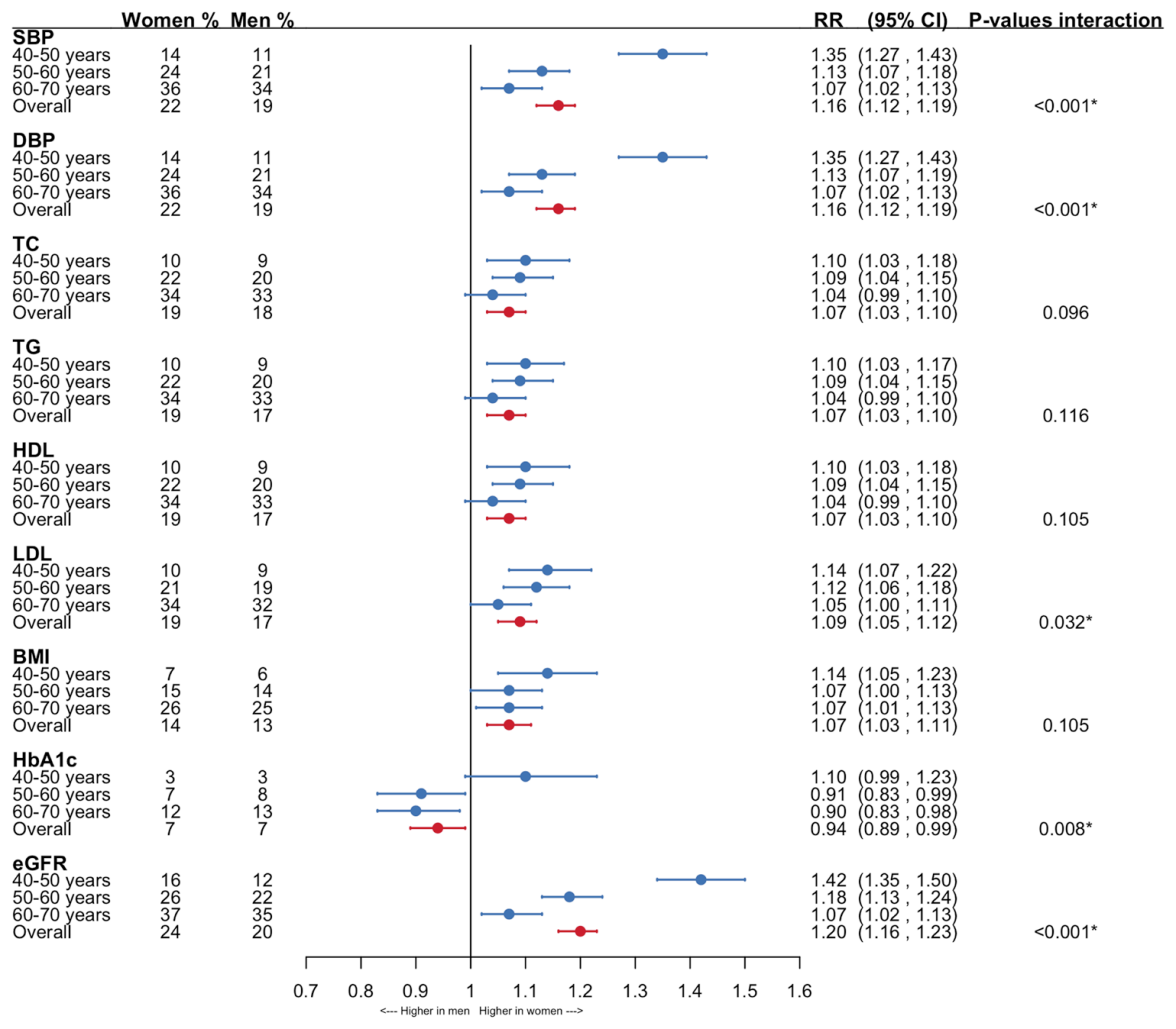
**Forestplot of the women-to-men risk ratios of measurement for CVD risk factors**. The analyses are adjusted for age and men are the reference category. The significant p-values show an interaction between age (as continuous variable) and sex. SBP = systolic blood pressure; DBP = diastolic blood pressure; TC = total cholesterol; TG = triglycerides; HDL = high-density lipoprotein cholesterol; LDL = low-density lipoprotein cholesterol; BMI = body mass index; HbA1c = glycated hemoglobin; eGFR = estimated glomerular filtration rate; RR = risk ratio; CI = confidence interval.

About two percent of both women and men were assessed for all nine CVD risk factors studied. Women were more likely than men to have a measurement for any individual risk factor, except for HbA1c. Compared with men, women were 16% (95% CI: 13–19) more likely to have a blood pressure measurement, 7% (95% CI: 3–10) more likely to have a TC, TG, or HDL-c measurement, 9% (95% CI: 5–12) more likely to have a LDL-c measurement, 7% (95% CI: 3–11) more likely to have a BMI measurement, 20% (95% CI: 16–23) more likely to have a eGFR measurement, and 6% (95% CI: -11–-1) less likely to have a HbA1c measurement (Figure 1). Analyses stratified by age showed that the sex differences attenuated with increasing age for SBP, DBP, LDL-c, HbA1c and eGFR.

### Risk factor levels

**Figure 2:**
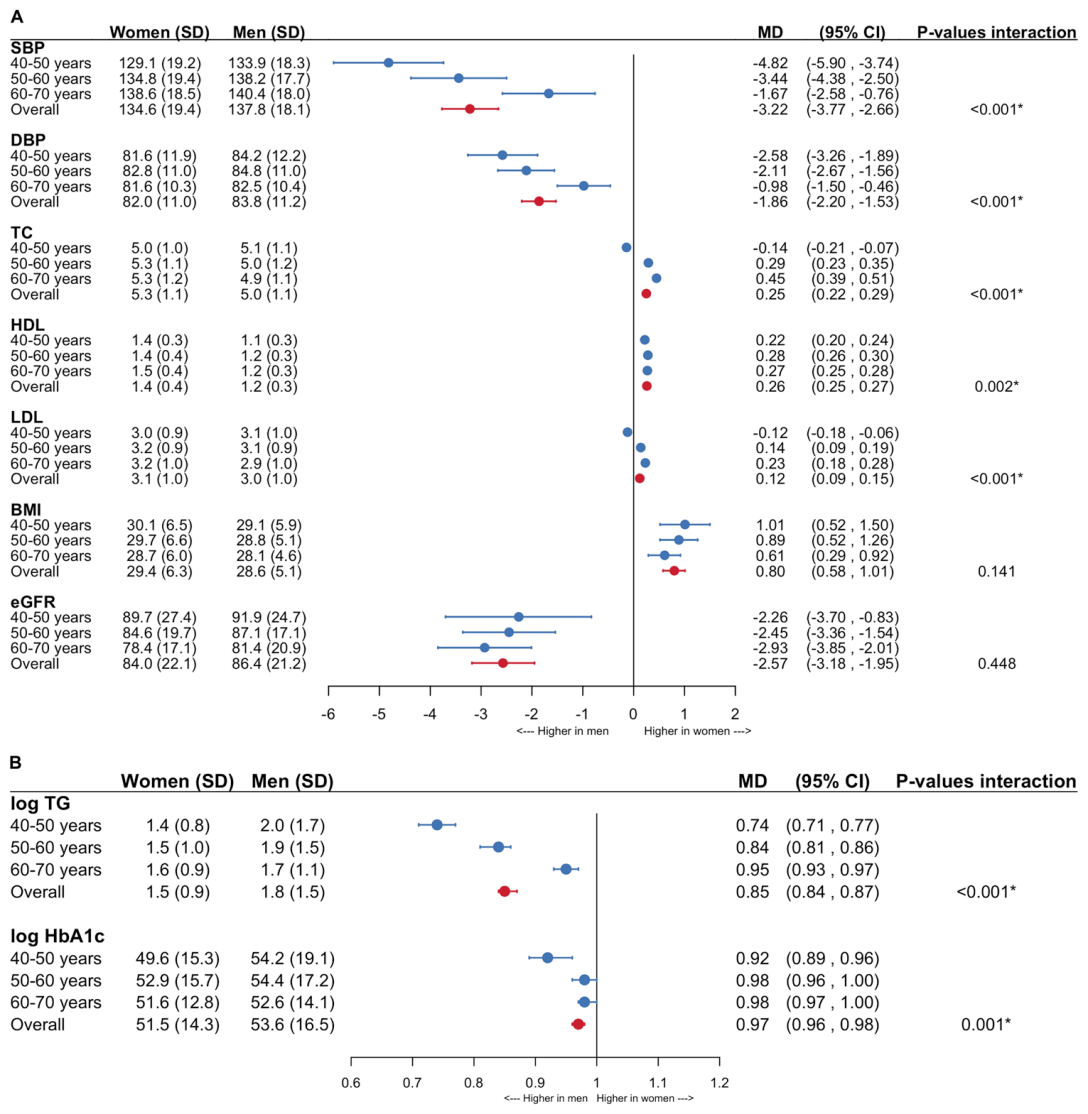
**Forestplots of the mean differences between women and men in the levels of cardiovascular risk factors**. The analyses are adjusted for age and men are the reference category. **A.** Non-transformed variables. **B.** Log-transformed estimates. SBP = systolic blood pressure; DBP = diastolic blood pressure; TC = total cholesterol; HDL = high-density lipoprotein cholesterol; LDL = low-density lipoprotein cholesterol; BMI = body mass index; eGFR = estimated glomerular filtration rate; TG = triglycerides; HbA1c = glycated hemoglobin; SD = standard deviation; MD = mean difference; CI = confidence interval.

Among the assessed individuals, women had lower values of SBP, DBP, eGFR, TG and HbA1c and higher values of TC, HDL, LDL-c and BMI than men (Figure 2). Age-stratified analyses showed smaller sex differences in levels of blood pressure, TG and HbA1c at older age.

### Treatment prescriptions

**Figure 3:**
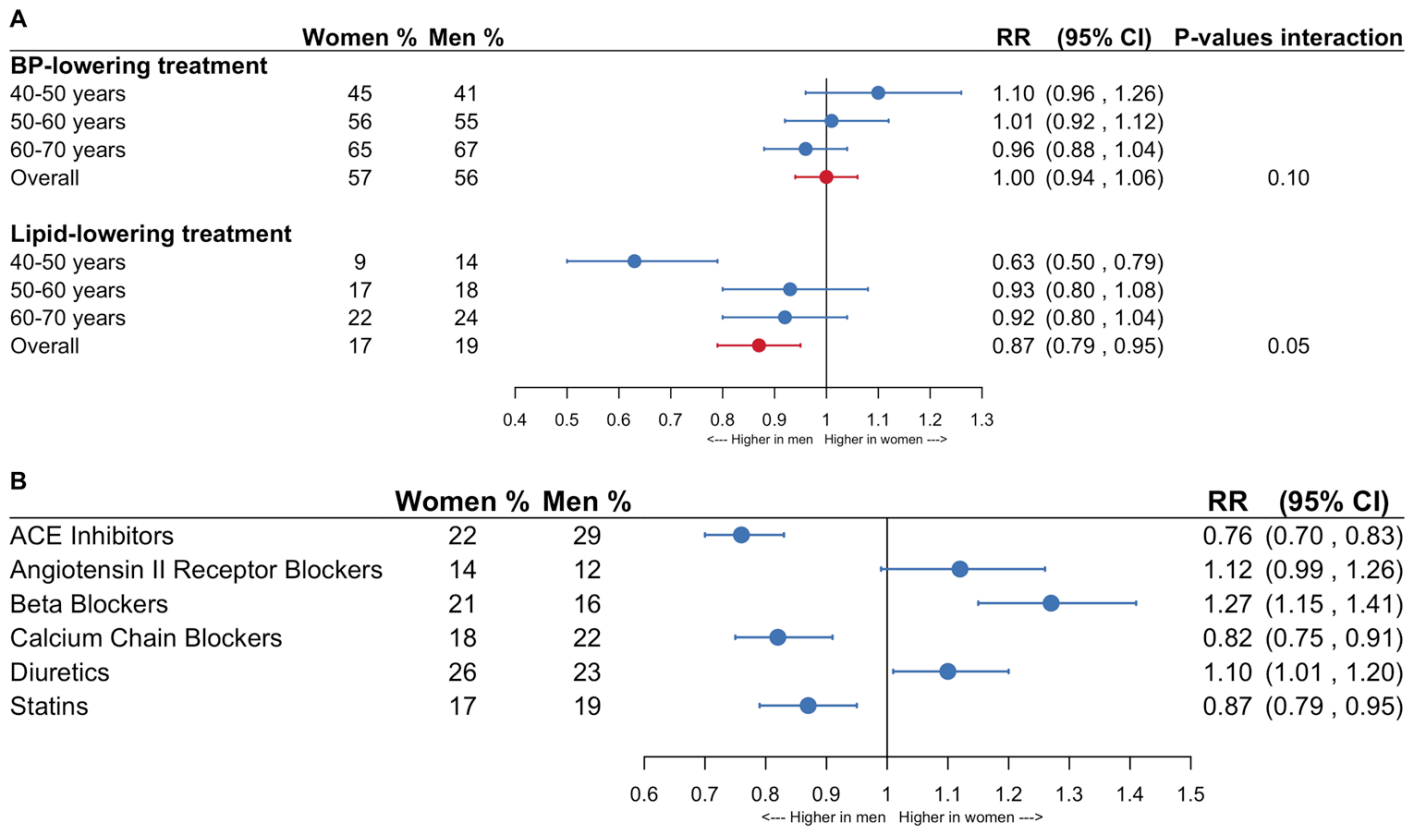
**Forestplots of the women-to-men risk ratios of being treated with antihypertensive drugs and lipid-lowering drugs**. The analyses were conducted in individuals with elevated risk factor levels. The analyses are adjusted for age and men are the reference category. **A.** General medication groups. **B.** Treatment subtypes. BP = blood pressure; ACE inhibitors = angiotensin converter enzyme inhibitors; RR = risk ratio; CI = confidence interval.

Ten percent of patients (n = 8,210, 4,190 women and 4,020 men) had a recording of elevated blood pressure levels and 12.4% (n = 10,405, 5,715 women and 4,690 men) had a recorded assessment of elevated LDL-c levels. Among the individuals with a recorded high blood pressure, 57% of women and 56% of men received antihypertensive treatment (RR 1.00, 95% CI: 0.94–1.06). The women-to-men risk ratios were smaller with older age but there was no significant interaction between age and sex (Figure 3a). The most and least prescribed antihypertensive medications were diuretics and ARB in women (26% and 14% of eligible women, respectively) and were ACE-i and ARB in men (29% and 12% of eligible men, respectively). Compared with men, women had a higher chance of being prescribed ARB (1.12, 0.99–1.26), beta blockers (1.27, 1.15–1.41) and diuretics (1.10, 1.01–1.20) and a lower chance of receiving ACE-i (0.77, 0.70–0.83) and CCB (0.82, 0.75–0.91) (Figure 3b).

For individuals with recorded high LDL-c levels, 17% of women and 19% of men were prescribed lipid-lowering treatment (0.87, 0.79–0.95). Women aged 40 to 50 years with elevated LDL-c levels had a lower chance of being prescribed treatment than similarly-aged men (0.63, 0.50–0.79), however, there was no statistically significant interaction between age and sex (Figure 3a).

### Risk factor control

**Figure 4:**
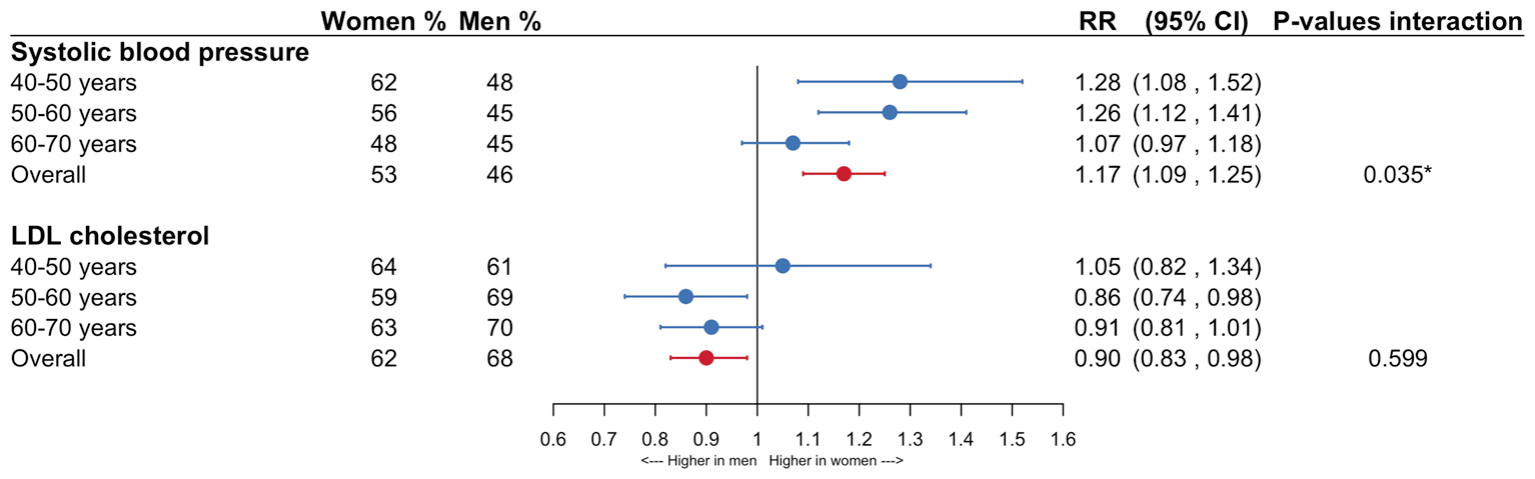
**Forestplot of the women-to-men risk ratios in the control of systolic blood pressure and LDL cholesterol**. The analyses were conducted in individuals with at least one prescription for antihypertensive or lipid-lowering medications in the 6 months prior to the CVD risk factor measurement. The analyses are adjusted for age and men are the reference category. LDL-c = low-density lipoprotein cholesterol; RR = risk ratio; CI = confidence interval.

Among individuals with a recent prescription for blood pressure-lowering drugs, 53% of women and 46% of men met the target levels for SBP. The corresponding RR was 1.17 (95% CI: 1.09–1.25). For lipid-lowering treatment, 62% of women and 68% of men met adequate values of LDL-c, resulting in a RR of 0.90 (95% CI: 0.83–0.98). There was a significant interaction between age and sex for blood pressure control, which attenuated with older age (Figure 4).

## Discussion

This large-scale study of 83,903 individuals registered in primary care shows that sex differences exist across the care continuum for the primary prevention of CVD, even in a well-established primary healthcare system such as in the Netherlands. Women were more likely to have their risk factors assessed as compared to men. While there was no sex difference in the prescription of antihypertensive medications, women were less likely than men to receive lipid-lowering medications. Once treated, women were more likely than men to meet target levels of SBP and less likely to meet target levels of LDL-c.

### Sex differences in risk factor assessment

Previous studies on risk factor measurement in primary prevention observed a lower chance for women to be measured as compared to men [10,11,19] or no difference between the sexes [20]. Some of the studies comparing primary and secondary prevention showed that the women-to-men risk ratios of the assessment for the CVD risk factors, increased in secondary prevention [19,20]. In our study, CVD risk factors were measured in a higher proportion of women than men. This was also observed in another study using the same data source [21].

A difference in the consultation habits between men and women could potentially explain the higher risk factor assessment in women. In the Netherlands, General practitioner (GP) practices use an opportunistic screening approach as opposed to a systematic screening program. As such, the assessment of CVD risk factors, to some extent, depends on the frequency of the patient’s visit to the GP [21]. On average, women consult the GP more often than men [22,23]. Wang et al. showed that sex differences in the consultation rates were the highest at younger ages, with differences persisting after accounting for reproductive-related consultations [23]. However, studies have found that sex differences in consultation rates disappeared when both sexes have similar underlying morbidities [23,24].

Our results showed that a higher proportion of young women (i.e., 40–50 years old) was assessed for blood pressure, LDL-c and eGFR, as compared to similarly-aged men. This was an unexpected result as the guidelines recommend the screening of CVD risk factors in women older than 50 years old [9].

In this study, we did not have access to the information on the number of contacts to the GP. Future work should investigate the significance of sex differences in consultation rates in primary prevention of CVD and their role in the patient’s clinical trajectory.

### Sex differences in risk factor levels

Previous studies have reported lower blood pressure [12,19,20] and higher cholesterol levels [12,19,20,25,26] in women as compared to men, which is also confirmed in the present study. Results are more contrasting for BMI [12,20,25,26]. In our study population, BMI levels were high (mean BMI 29.0 kg/m^2^), which may be because individuals with elevated BMI values are more likely to have their BMI measured in primary care. At all ages, women had higher BMI levels than men. BMI was also measured more often in women, which is consistent with the hypothesis that BMI is more likely to be measured when abnormal. Also, in line with the opportunistic screening approach, a high BMI might act as trigger for the primary care worker to perform a cardiovascular risk profile. While BMI is related to adverse risks for CVD, body fat distribution gives a more accurate portrayal of the detrimental effects of fat on risk of CVD. Women’s adipose profile is known to change over time, with more accumulation of abdominal fat, thus increasing their risk for CVD [27,28].

### Sex differences in treatment prescription

Overall, our study showed low treatment rates for both women and men. Though these rates are similar in other studies, efforts should be made to ensure optimal treatment in individuals at increased risk of CVD.

We showed that women were less likely to have a prescription for lipid-lowering drugs than men, which is in line with previous studies in the primary prevention of CVD [10,11,26,29,30]. One study showed heterogeneity by age; women aged 35 to 54 were less likely to be prescribed medications, while women aged ≥ 65 were more likely to be prescribed medications as compared to their male counterparts [10]. Although more women die from CVD than men, risk estimates are lower in women than in men, at any given age [8]. Therefore, men may be more likely to be considered to benefit from drug therapy and to meet target recommendations for initiating a lipid-lowering treatment [26]. Accordingly, the sex-specific risk score tables used in clinical practice to assess CVD risk are a possible explanation for our observations. Although smoking is an important driver of cardiovascular risk, especially in women, the absence of information on smoking status in this study precluded the calculation of a CVD risk score. The lower prescription rates for lipid-lowering medications in young women found in our study could also be explained by a lack of awareness and implicit gender biases (i.e., unconscious attitude or stereotype that can lead to unequal treatment of people based on their gender).

Our findings, in line with several studies, observed no sex difference in the prescription for antihypertensive medications [20,26,30], while other studies found that women were more likely to be treated than men [11,31].

Although no sex difference was observed for the prescription of antihypertensive medications as a whole, a significant variability was observed across the different subtypes. A systematic review by Zhao et al. involving more than 2 million participants worldwide (of which 28% were women) showed that women were more likely to be on diuretics and less likely to be on ACE-i as compared to men [30]. This result has already been observed in previous studies [32,33] and, to this day, remains largely unexplained.

### Sex differences in risk factor control

Our analyses identified two tendencies in the sex differences of CVD risk factor control that have previously been observed in studies involving patients with established CVD [12,34]. First, treated women reached less often LDL-c control than men, and second, women reached more often blood pressure control than men. Multiple studies have confirmed the effectiveness of statins in primary prevention in women, as well as in men [35,36,37]. Therefore, the observed lower achievement of LDL-c target levels in treated women compared to men is unlikely to be due to sex differences in statin efficacy. An alternative explanation could stem from sex differences in treatment usage. Nanna et al. investigated the reasons for statin undertreatment in women in the USA and reported that women were more likely to decline and discontinue statin therapy and were less likely to believe in the safety and effectiveness of statins, than men [29]. Besides, female sex has been suggested as risk factor for statin-induced myalgia [38], although, this has been called into question by the results of a recent study that showed that the risk of statin-induced muscle pain in trial participants is quite low [39]. Taken together, these reasons could contribute to explain the lower adherence to statins in women seen in the literature [38,40,41]. A lower adherence to statins in women than in men would be an explanation for the observed sex differences in the control of LDL-c.

In our study, there was no sex difference in the prescription of antihypertensive drugs, but treated women met more often the blood pressure targets than treated men. One potential explanation is that women might need lower doses of blood pressure-lowering medications than men to achieve the treatment target. Santema et al. investigated sex differences in the optimal dose of ACE inhibitors or ARBs and beta blockers in patients with heart failure with reduced ejection fraction (HFrEF) and showed that women had 30% lower risk at only 50% of the recommended doses, with no further decrease in risk at higher dose levels [42]. Reasons why women respond differently to drugs than men include lower body weight and organ size, higher percentage of body fat, lower renal clearance, slower gastric emptying time and gastric pH, lower plasma volume and physiological changes during the menstrual cycle [43]. It is essential to address sex differences in the optimal dose of CVD medications, as inadequate doses may lead to adverse drug reactions that negatively affect medication adherence and long-term prognosis [44].

### Strengths and limitations

The main strength of the study is the use of routine clinical data from a large dynamic population, whose demographic characteristics are representative for the Dutch population [17].

This study also has several limitations. The quality of the data depends on the accuracy of recording of the GP. When not properly recorded, misclassification of the outcome can occur as well as data entry errors that can lead to loss of information during the preprocessing of the data. For example, it is possible that the blood pressure of some individuals was measured at the GP practice but was not reported in the EHR, or not reported in the appropriate diagnostic record. Moreover, informative missingness could have introduced some bias. For example, the absence of a risk factor measurement in an individual could imply that the measurement was normal. Therefore, without accounting for missing values, the average levels of the CVD risk factors in the study might be biased. However, both data entry errors and informative missingness are thought to be non-differential between men and women and are unlikely to have influenced the main findings of this study. Since the database only captures primary care data, medications prescribed in secondary and tertiary care were not included. Likewise, individuals with a history of CVD that was not recorded at the GP practice might have been included in the study population. Measurements of blood pressure and LDL-c were frequently not available, which might also have impacted the analyses of risk factor treatment and control. As smoking was not well documented, we were not able to calculate a risk score. We therefore investigated treatment prescriptions in individuals with an indication for treatment based on measurements alone. Moreover, eligibility for treatment was based on the value of a single measurement, while clinical practice endorses a more comprehensive approach. Future work looking at sex differences in the primary prevention of CVD should take heed of including the predicted CVD risk score. Although this study looked at nine CVD risk factors, we had no data on risk factors such as smoking, diet and sedentary lifestyle. If available, this information is usually found in the free-text. However, only structured data was used in our study. Additionally, information on ethnicity was not available in this study. Finally, information on medication was based on prescribed and not dispensed medication data.

## Conclusions

Sex differences exist at several stages of the care continuum for the primary prevention of CVD in a well-organised primary care setting, and to some extent, vary by age. The reasons behind these differences and implications for disease outcomes are two areas of research that require undivided attention.

## Additional File

The additional file for this article can be found as follows:

10.5334/gh.1284.s1 Supplementary File. Supplementary Figures 1 to 3 and Supplementary Tables 1 and 2.
