# Supplementary material for: Sex Differences in the Primary Prevention of Cardiovascular Diseases in a Dutch Primary Care Setting
Source: Glob Heart. 2024 Jan 19;19(1):6. doi: 10.5334/gh.1284 (PMC10798167; doi:10.5334/gh.1284)
Supplement: Supplementary File. — Supplementary Figures 1 to 3 and Supplementary Tables 1 and 2. [file gh-19-1-1284-s1.pdf]

Supplementary Figure 1.

REPEAT-IT Graphical depiction of the study design: assessment and levels of risk factors.

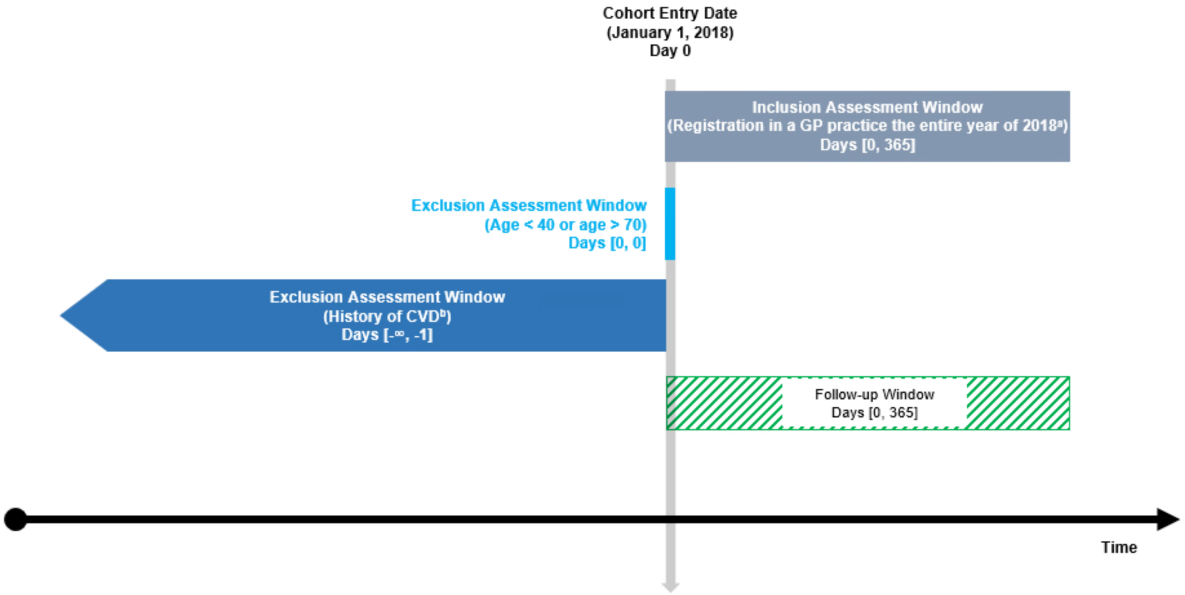

a. Inclusion to the study population possible only if the GP practice attributed data to JGPN during the study period.
b. The following ICPC codes were used in the definition of cardiovascular diseases: K74, K7401, K7402, K75, K76, K7601, K7602, K77, K89, K8901, K9003, K9201, K9901. This exclusion criteria applies in absence of measurement for a CVD risk factor recorded in 2018. In presence of a measurement recorded in 2018, we excluded individuals with a CVD event recorded in the EHR before the date of the first measurement.

Supplementary Figure 2.

REPEAT-IT Graphical depiction of the study design: risk factors treatment.

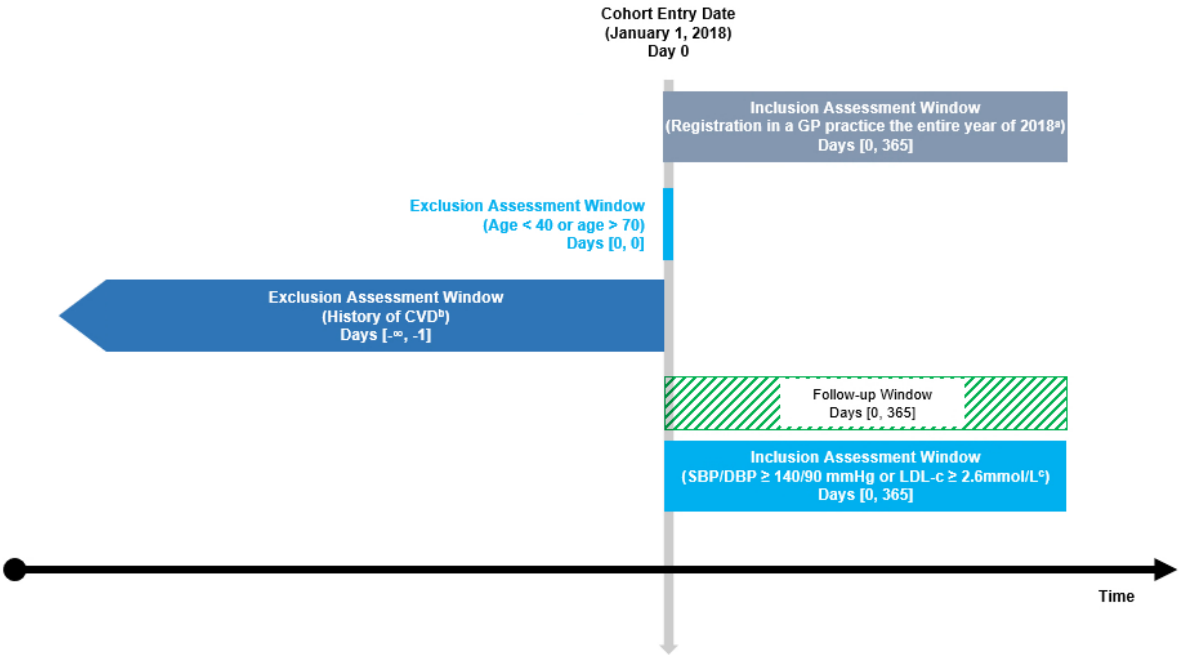

a. Inclusion to the study population possible only if the GP practice attributed data to JGPN during the study period.
b. The following ICPC codes were used in the definition of cardiovascular diseases: K74, K7401, K7402, K75, K76, K7601, K7602, K77, K89, K8901, K9003, K9201, K9901. This exclusion criteria applies in absence of measurement for a CVD risk factor recorded in 2018. In presence of a measurement recorded in 2018, we excluded individuals with a CVD event recorded in the EHR before the date of the first measurement.
c. Risk factor treatment was assessed in individuals with a Systolic Blood Pressure (SBP) / Diastolic Blood Pressure (DBP) ≥ 140/90 mmHg or with Low Density Lipoprotein cholesterol (LDL-c) ≥ 2.6mmol/L. The risk factor levels were assessed with the value of the first measurement in 2018.

Supplementary Figure 3.

REPEAT-IT Graphical depiction of the study design: risk factor control.

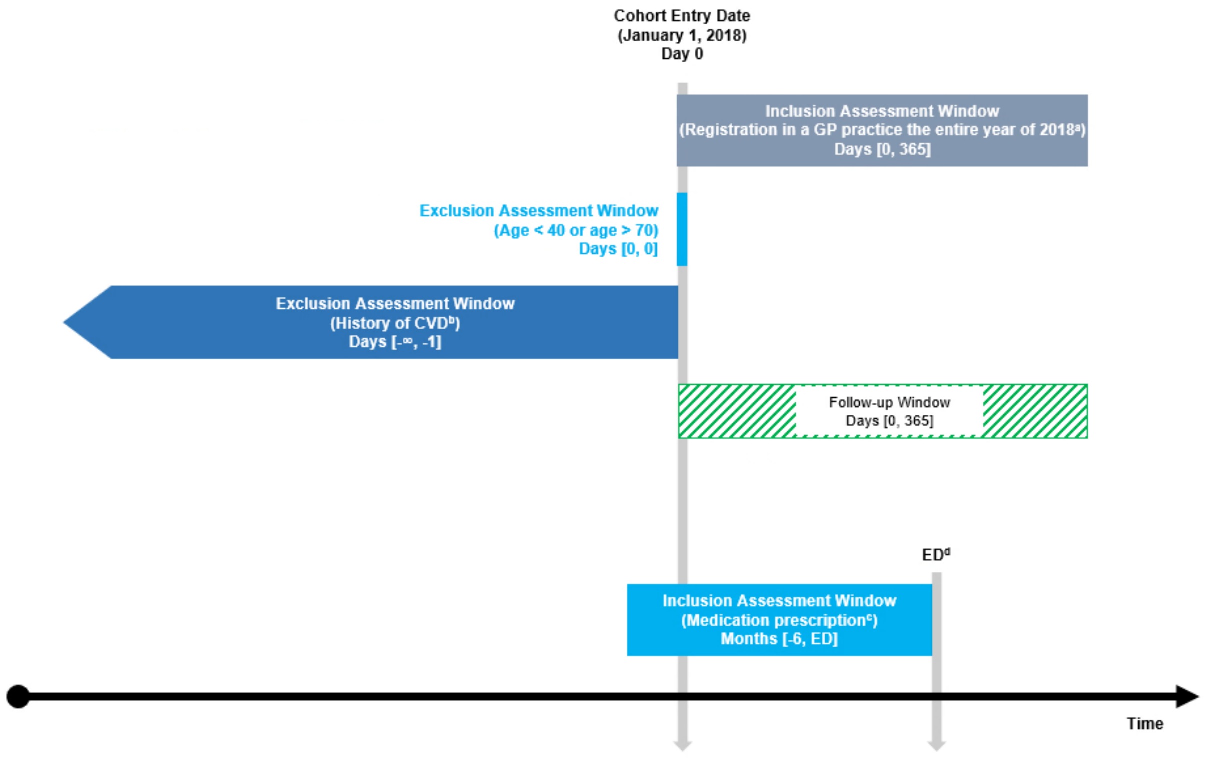

a. Inclusion to the study population possible only if the GP practice attributed data to JGPN during the study period.
b. The following ICPC codes were used in the definition of cardiovascular diseases: K74, K7401, K7402, K75, K76, K7601, K7602, K77, K89, K8901, K9003, K9201, K9901. This exclusion criteria applies in absence of measurement for a CVD risk factor recorded in 2018. In presence of a measurement recorded in 2018, we excluded individuals with a CVD event recorded in the EHR before the date of the first measurement.
c. The following classes of medications were included (ATC code): antihypertensives (C02*), diuretics (C03*), beta-blockers (C07*), calcium-chain blocker (C08*), ACE inhibitors (C09A* and C09B*), ARB (C09C* and C09D*), HMG CoA reductase inhibitors (C10AA), Fibrates (C10AB), Bile acid sequestrants (C10AC), nicotinic acid and derivatives (C10AD).
d. ED = event date = date of the first risk factor measurement in 2018.

Supplementary Table 1.

Definitions and codes for medical history and drug prescriptions.

**ICPC codes for the definition of cardiovascular history**	
K74	Angina pectoris
K7401	Unstable angina pectoris
K7402	Stable angina pectoris
K75	Acute myocardial infarction
K76	Other/chronic ischemic heart disease
K7601	Coronary sclerosis
K7602	Myocardial infarction (>4 weeks ago)
K77	Heart failure
K89	Transient ischemic attack
K9003	Stroke / cerebrovascular accident
K9201	Peripheral artery disease
K9901	Aortic aneurysm
**ATC codes for the definition of antihypertensive medications**	
C02	Antihypertensive
C03	Diuretics
C07	Beta blockers
C08	Calcium channel blockers
C09A/C09B	ACE-i
C09C/C09D	ARB
**ATC codes for the definition of lipid-lowering medications**	
C10AA	HMG CoA reductase inhibitors
C10AB	Fibrates
C10AC	Bile acid sequestrants
C10AD	Nicotinic acid and derivatives

With ICPC = International Classification of Primary Care ; ATC = anatomical therapeutic chemical classification ; ACE inhibitors = Angiotensin-converting enzyme inhibitors ; ARB = Angiotensin II receptor blockers.

Supplementary Table 2.

Percentages of individuals with at least one medication prescription in the 6 months prior to the measurement of systolic blood pressure or low-density lipoprotein cholesterol.

	Overall	Women	Men
**N***	83 903	42 262	41 641
**Medications (%)**			
Lipid-lowering drugs	4.2	4.1	4.3
Statins	4.2	4.1	4.4
Antihypertensive drugs	7.8	8.6	7.1
ACE inhibitors	3.2	3.0	3.5
ARB	1.8	1.99	1.6
Beta blockers	2.6	3.1	2.0
Calcium chain blockers	2.2	2.2	2.3
Diuretics	3.1	3.5	2.6

With ACE inhibitors = Angiotensin-converting enzyme inhibitors ; ARB = Angiotensin II receptor blockers.
* N refers to the number of individuals included in the study and only includes individuals registered in 2018.
